# Isotope Effects in the Predissociation of Excited States of N_2_^+^ Produced by Photoionization of ^14^N_2_ and ^15^N_2_ at Energies Between 24.2 and 25.6 eV

**DOI:** 10.3389/fchem.2019.00222

**Published:** 2019-04-12

**Authors:** Helgi R. Hrodmarsson, Roland Thissen, Danielle Dowek, Gustavo A. Garcia, Laurent Nahon, Thomas R. Govers

**Affiliations:** ^1^Synchrotron SOLEIL, L'Orme des Merisiers, Saint-Aubin BP 48, Gif-sur-Yvette, France; ^2^Laboratoire de Chimie Physique, Université Paris-Sud, Orsay, France; ^3^Institut des Sciences Moléculaires, Université Paris-Sud, Orsay, France; ^4^Soleil Synchrotron, Paris, France

**Keywords:** photoionization, nitrogen, predissociation, charge exchange, helium, TPEPICO

## Abstract

Photoelectron/photoion imaging spectrometry employing dispersed VUV radiation from the SOLEIL synchrotron has been used to study the predissociation of N_2_^+^ states located up to 1.3 eV above the ion's first dissociation limit. Branching ratios for unimolecular decay into either N_2_^+^ or N^+^ were obtained by measuring coincidences between threshold electrons and mass-selected product ions, using a supersonic beam of either ^14^N_2_ or ^15^N_2_ as photoionization target. The results confirm that predissociation of the ${\text{C}}^{2}{\text{Σ}}_{\text{u}}^{+}$ state of ^14^N_2_^+^ is faster than emission to the electronic ground-state by a factor 10 or more for all vibrational levels v′ ≥ 3, while for ^15^N_2_^+^ the two decay modes have comparable probabilities for the levels v′ = 3, 4, and 5. In contrast, no significant isotope effect could be observed for the other states of N_2_^+^ identified in the photoelectron spectrum. For both ^14^N_2_^+^ and ^15^N_2_^+^ isotopologues all vibrational levels of these other states decay to an extent of at least 95% by predissociation.

## Introduction

The properties of the excited states of N_2_^+^ in the neighborhood of its first dissociation limit are of relevance to thermal charge exchange between He^+^ and N_2_ and its possible role in the escape of helium from the earth's atmosphere. For such a reaction to contribute to the loss of helium, its exothermicity should be high enough to impart to the He product a kinetic energy of about 2.5 eV. The identity and relative importance of the primary charge-transfer channels need to be known to evaluate that possibility (see Lie-Svendsen et al., 1992).

**Figure 1:**
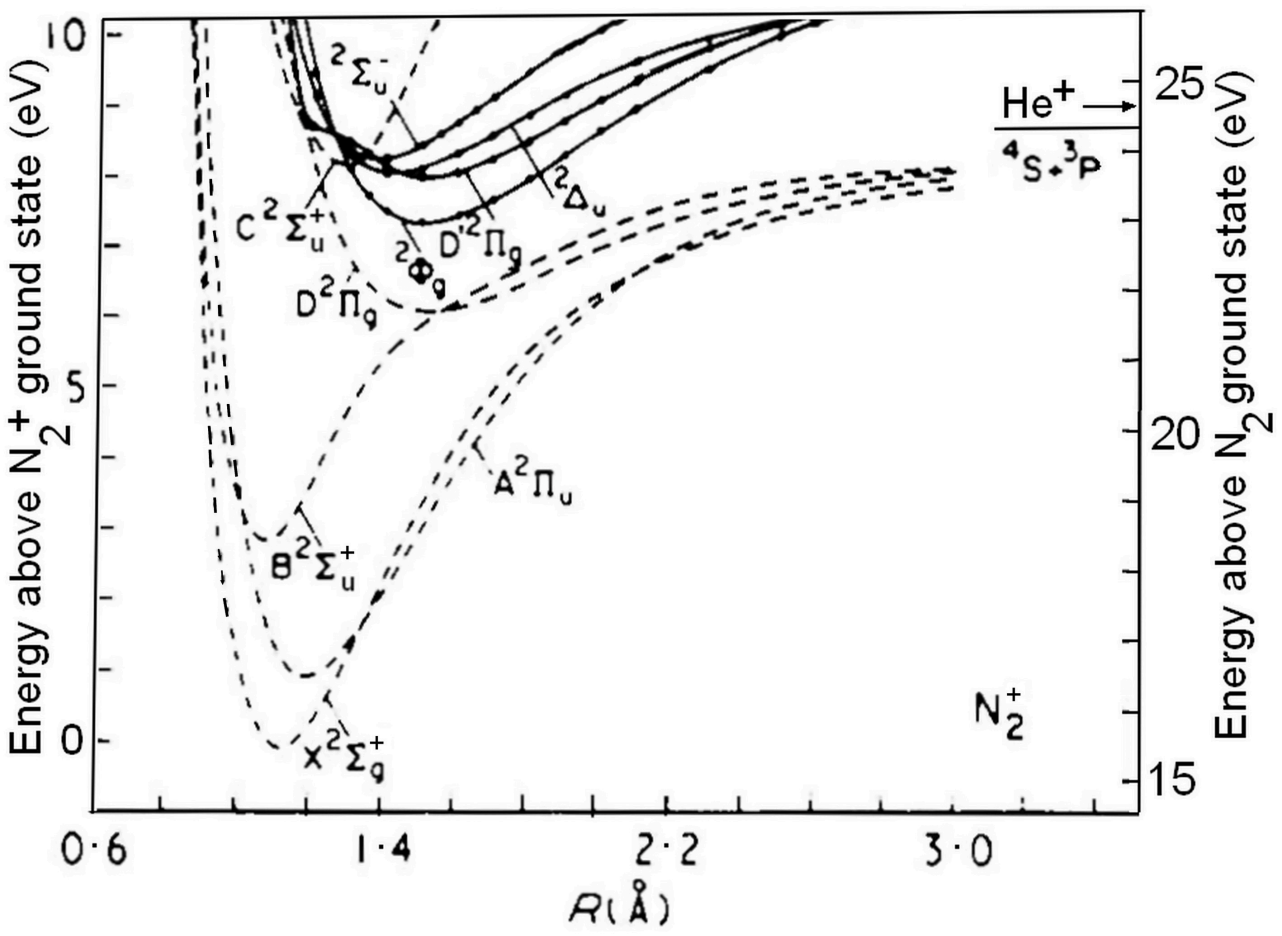
Potential-energy curves for doublet states of N_2_^+^ mentioned in the text. The left-hand ordinate indicates the energy with respect to the N_2_^+^(X ^2^${\text{Σ}}_{\text{g}}^{+}$) ground-state, the ordinate at the right gives the energy with respect to the ground-state of the neutral molecule. The arrow indicates the recombination energy of He^+^: 24.5874 eV. Adapted from Cartwright and Dunning (1975), whose permission is gratefully acknowledged.

Among the doublet states, illustrated in Figure 1, the ${\text{C}}^{2}{\text{Σ}}_{\text{u}}^{+}$ state in particular has been the subject of many experimental and theoretical investigations (van de Runstraat et al., 1974; Paulus et al., 2016, and references therein). Its lower vibrational levels, v′ ≤ 2, lie below the ground-state N^+^(^3^P) + N(^4^S) asymptote and fluorescence to the electronic ground-state, N_2_^+^ (${\text{C}}^{2}{\text{Σ}}_{\text{u}}^{+}$, v′) → X (${}^{2}{\text{Σ}}_{\text{g}}^{+}$, v″), is their only known unimolecular decay path. This emission occurs at wavelengths between 127 and 223 nm, and is known as the second negative system of N_2_^+^ (Lofthus and Krupenie, 1977). The levels v′ ≥ 3, on the other hand, can also decay by unimolecular predissociation into ground-state atomic fragments. Spectroscopically, the onset of predissociation is characterized by a weakening in the C → X fluorescence, and a decreasing N_2_^+^ (${\text{C}}^{2}{\text{Σ}}_{\text{u}}^{+}$, v′) lifetime. In mass spectrometry the predissociation will manifest itself by the production of N^+^ ions in competition with that of N_2_^+^.

The competition between C-state predissociation and fluorescence has first been experimentally quantified by the analysis of the vibrationally resolved C → X emission spectrum. In the work by van de Runstraat et al. (1974) and Govers et al. (1975), the C (v′) levels were populated under conditions where their relative populations should reflect a “vertical” ionization process, so that the vibrational fluorescence intensities could be predicted in the absence of predissociation. Comparison with the relative intensities that were actually observed made it possible to determine the ratio between the predissociation- and fluorescence probabilities, A_pred_ (v′)/A_em_ (v′), for each of the C-state vibrational levels v′ = 3–8. Electron- and ion-impact experiments using a room-temperature nitrogen target were conducted for the three isotopologues: ^14^N_2_, ^14^N^15^N, and ^15^N_2_. It was found that the predissociation probability generally increases with increasing vibration, and that it is subject to a strong isotope effect. The quantitative uncertainty in this approach stems largely from the difficulty to predict the initial population of C-state vibrational levels, as will be discussed further below. Two alternate methods can be used to avoid this difficulty. The first one is to measure the individual lifetimes τ(v′), since:

(1) $$\begin{array}{l} 1/\tau \left({\text{v}}^{\prime }\right)={\text{A}}_{\text{em}}\left({\text{v}}^{\prime }\right)\left[1+{\text{A}}_{\text{pred}}\left({\text{v}}^{\prime }\right)/{\text{A}}_{\text{em}}\left({\text{v}}^{\prime }\right)\right] \end{array}$$

where the emission rate A_em_ (v′) varies little with vibrational number and can be measured for the non-predissociated levels. Such measurements have been reported by Erman (1976) for ^14^N_2_^+^ (C, v′), with v′ = 0–5. They demonstrated a 20-fold decrease in lifetime between v′ = 2 and v′ = 3, but did not confirm the further decrease with increasing vibration. The experiment is difficult because of the low intensities of fluorescence from v′ ≥ 3, and would be easier to carry out with ^14^N^15^N or ^15^N_2_.

The other method, used in the present work, is to concurrently measure mass-selected ion yields, I(N_2_^+^) and I(N^+^), upon selective ionization of a specific (C, v′) level, to directly obtain the respective branching ratios:

(2) $$\begin{array}{lll} \text{BR}\left({\text{v}}^{\text{′}},{\text{N}}_{2}^{+}\right) & = & \text{I}\left({\text{v}}^{\text{′}},{\text{N}}_{2}^{+}\right)/\left[\text{I}\left({\text{v}}^{\prime },{\text{N}}_{2}^{+}\right)+\text{I}\left({\text{v}}^{\prime },{\text{N}}^{+}\right)\right];\\ \text{BR}\left({\text{v}}^{\text{′}},{\text{N}}^{+}\right) & = & 1-\text{BR}\left({\text{v}}^{\text{′}},{\text{N}}_{2}^{+}\right) \end{array}$$

from which:

(3) $$\begin{array}{l} {\text{A}}_{\text{pred}}\left({\text{v}}^{\prime }\right)/{\text{A}}_{\text{em}}\left({\text{v}}^{\prime }\right)=\left[1-\text{BR}\left({\text{v}}^{\text{′}},{\text{N}}_{2}^{+}\right)\right]/\text{BR}\left({\text{v}}^{\prime },{\text{N}}_{2}^{+}\right) \end{array}$$

The TPEPICO (threshold photoelectron/photoion coincidence) technique using a photoelectron/photoion imaging spectrometer is well-suited for this purpose.

## Methods

The experiments were performed on the DESIRS VUV beamline (Nahon et al., 2012) of the French synchrotron facility SOLEIL. Horizontally polarized VUV light emitted from an undulator was dispersed by a 6.65 m normal incidence monochromator equipped with a 4,300 grooves/mm grating. Its exit slit was set at 400 μm, providing a photon energy bandpass at 24.57 eV of 2.5 meV full width at half maximum (“fwhm”). The photon beam crossed a supersonic molecular beam of nitrogen in the SAPHIRS end station (Tang et al., 2015), which houses the DELICIOUS III double imaging spectrometer (Garcia et al., 2013). It is composed of a velocity map imaging analyzer of photoelectrons and a modified Wiley-McLaren time-of-flight ion imaging device, operated in coincidence. Electrons and ions are extracted and accelerated vertically in opposite directions by a constant electric field, perpendicular to the plane defined by the molecular beam and the photon beam, and they are detected in coincidence by means of delay-line based position sensitive channel plate detectors. The extraction field in the source region was set at 18 V/cm, ensuring full collection of photoelectrons and photoions with kinetic energies up to about 0.8 eV. The electron energy resolution under these conditions is about 3 to 4% and the ion mass resolution Δm/m ≈ 1/350. Unsubstituted nitrogen was N60 grade from Air Liquide, while the ^15^N_2_ isotopolog was supplied by Aldrich, with a ^14^N_2_ content of <2%.

In the present experiments, the stagnation pressure of the 30 μm diameter nozzle was 0.7 Bar, and the supersonic beam was defined by two consecutive skimmers, the first of which, with a diameter of 1 mm, was located 10 mm downstream from the nozzle, and the second, with a diameter of 2 mm, at a distance of 25 mm. The crossing point with the photon beam was located at 5 cm from the nozzle, far downstream from the onset of freezing of the rotational distribution of the expanding nitrogen. An estimate of the rotational population can thus be obtained from the data obtained by Mori et al. (2005) who report results for a pressure x nozzle diameter product of 15 Torr.mm, close to our value of 16 Torr.mm. Under these conditions, the rotational distribution has its maximum at *N* = 3, and 90% of the N_2_ molecules have a rotational quantum number of *N* = 7 or less. In comparison, the average *N* for room-temperature nitrogen is 9. Downstream from rotational freezing, Mori et al. (2005) found the limiting population of the lower rotational levels to be characterized by a rotational “temperature” between 30 and 40 K. In comparison, the present N_2_^+^ velocity distribution extracted from the ion position and TOF (see Tang et al., 2015) was consistent with a beam translational temperature close to 50 K.

The absence of significant mass dependence of the ion detection efficiency was verified by measuring the ratio of the threshold photoelectron/photoion coincidences to the corresponding number of electron starts, which yields the absolute ion detection efficiency as outlined by Brehm et al. (1995). At the threshold for He ionization, the amu = 4 detection efficiency was found to be 28 ± 1%, while at the thresholds for ionization to ^14^N_2_^+^ and ^15^N_2_^+^, the mass 28 and mass 30 detection efficiencies were found to be 31 ± 1% and 29 ± 1%, respectively. As the response of the micro channel plates depends primarily on the ion's impact velocity, the close similarity between the data obtained for mass 4 and for masses 28 and 30 indicates that mass discrimination between N_2_^+^ and N^+^ can be neglected. As indicated above, discrimination resulting from the kinetic energy of the N^+^ fragments is negligible up to values of about 0.8 eV, while the highest value reached in this work is only 0.66 eV. Branching ratios for N_2_^+^ and N^+^ production can thus directly be extracted from their relative coincidence rates in the TPEPICO spectra.

## Results

The photon energy calibration and bandwidth were verified with a He beam as target by measuring the onset for coincidences between He^+^ ions and photoelectrons observed upon scanning the photon energy between 24.56 and 24.62 eV in 1 meV steps. The threshold was found at a nominal value of 24.5865 eV, which agrees within 1 meV with the published value of 24.5874 eV (Kandula et al., 2010). The spectra shown in this paper have been corrected for this offset. The photon energy width was measured as 2.5 meV, fwhm. Throughout this paper we used the conversion factor 1 eV = 8065.544005 cm^−1^ (Mohr et al., 2016).

**Figure 2:**
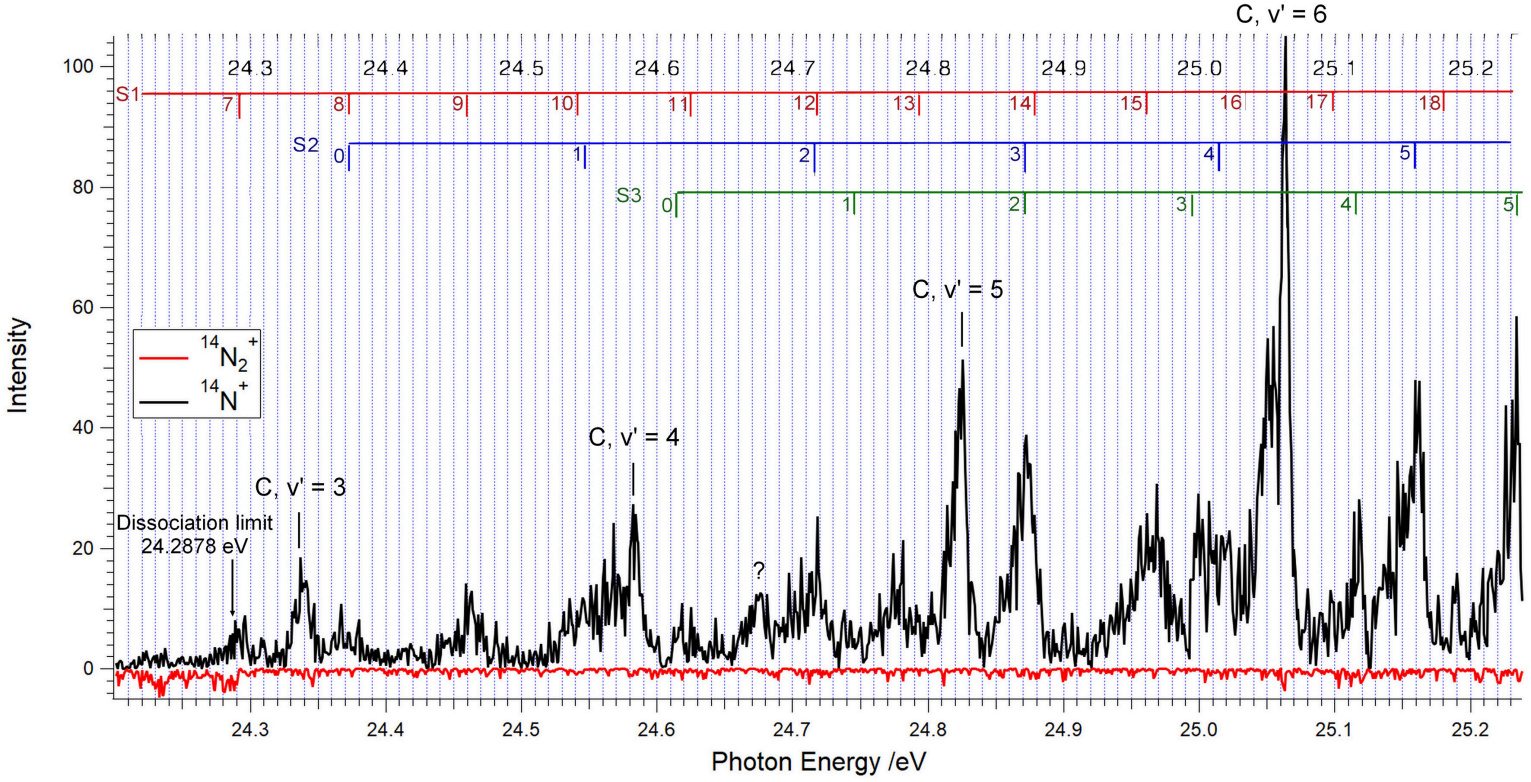
TPEPICO spectrum for ^14^N_2_ between 24.0 and 25.24 eV showing the vibrational levels v′ = 3–6 of the C ^2^${\text{Σ}}_{\text{u}}^{+}$ state of ^14^N_2_^+^. The photon energy was scanned in 1 meV increments, and the electron-ion coincidences were integrated during 40 s after each step. Coincidences with ^14^N^+^ ions are indicated in black, those with ^14^N_2_^+^ are shown upside down, in red.

Figure 2 shows our ^14^N_2_ TPEPICO spectrum obtained by selecting coincidences with electrons having energies between 0 and 5 meV. The black trace shows the N^+^ coincidence rate, and the red trace, drawn upside down, corresponds to N_2_^+^ coincidences.

**Figure 3:**
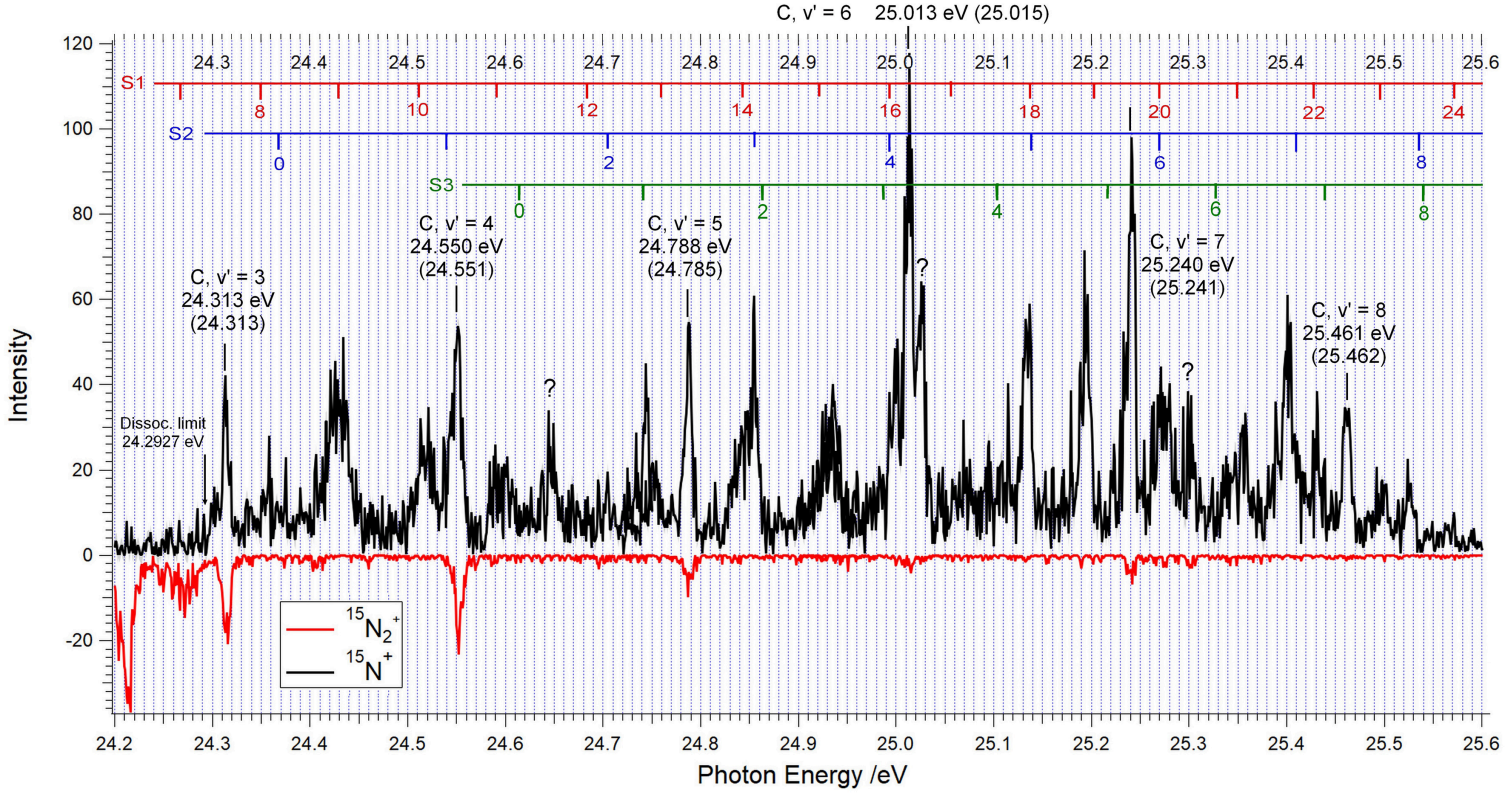
TPEPICO spectrum for ^15^N_2_ between 24.2 and 25.6 eV showing the vibrational levels v′ = 3–8 of the C ^2^${\text{Σ}}_{\text{u}}^{+}$ state of ^15^N_2_^+^ at the energies as labeled. The photon energy was scanned in 1 meV increments, and the electron-ion coincidences were integrated during 40 s after each step. Coincidences with ^15^N^+^ ions are indicated in black, those with ^15^N_2_^+^, drawn upside down, are in red.

One readily identifies the vibrational levels v′ = 3, 4, 5, and 6 of the ^14^N_2_^+^ C-state, as indicated. The marks and labels in the top of Figure 3 correspond to the vibrational progressions called S1, S2, and S3 in the TPES (threshold photoelectron spectrum) measured by Yoshii et al. (1997). The vibrational quantum numbers are positioned at the photon energies obtained from their tabulated wavelengths. The S1 progression corresponds to the D′ ${}^{2}{\text{Π}}_{\text{g}}$ state of Figure 1, as will be discussed further below. The S2 and S3 progressions have been assigned to the ${}^{2}{\text{Σ}}_{\text{u}}^{-}$ and ${}^{2}\Delta_{\text{u}}$ states shown in Figure 1, respectively (Yoshii et al., 1997).

The C, v′ = 5 level has a fwhm of 10 meV, somewhat larger than the compounded instrumental width of 6 meV. Yoshii et al. (1997), with a slightly better resolution and a supersonic molecular beam target, measured a threshold photoelectron width of 7.5 meV. Yencha et al. (2014), who used a static target cooled to 77 K and an instrumental resolution of 18 meV, obtained a halfwidth of about 32 meV. From the data reported by Merkt and Guyon (1993), one can evaluate the width of the rotational band structure for a 300 K gas target to be of the order of 20–30 meV. The narrowness of the C, v′ = 5 peak observed by Yoshii et al. (1997) and in the present experiments indicates that the supersonic beams used in these two investigations ensure a target rotational distribution significantly narrower than that of a static gas at 77 K, in agreement with the findings of Mori et al. (2005) mentioned above.

Table 1 compares the energy positions of the spectral features of Figure 1 with those obtained from the analysis of the ^14^N_2_^+^ (${\text{C}}^{2}{\text{Σ}}_{\text{u}}^{+}$, v′) → X (${}^{2}{\text{Σ}}_{\text{g}}^{+}$, v″) emission spectrum by Joshi (1996b), the ZEKE measurements of Merkt and Guyon (1993) and the TPES spectrum of Yoshii et al. (1997). The assignments are listed in the right-hand column, with the vibrational number indicated in parenthesis. Our energy peaks for the C-state agree within 1 meV with those obtained from the C → X emission bandheads measured by Joshi (1996b) and the ^14^N_2_ ionization threshold of Huber and Jungen (1990). The feature observed at 24.677 eV is also seen in the TPES spectrum of Yoshii et al. (1997), but is as yet unassigned. The S1(13) peak, which Yoshii et al. (1997) situate at 24.793 eV is reported at 24.788 eV by Baltzer et al. (1992) and at 24.780 eV by Yencha et al. (2014), which substantiates the assignment of the peak we observed at 24.779 eV.

**Table 1:** Spectral features observed in the TPEPICO spectrum of ^14^N_2_ between 24.2 and 25.24 eV (Figure 1).

**These expts**	**Joshi**	**Merkt & Guyon**	**Yoshii et al**.	**Assignment**
24.292			24.292	S1(7)
24.337	24.338	24.337	24.336	C(3)
24.370			24.373	S1(8) or S2(0)
24.461			24.460	S1(9)
24.56 approx.		24.541	24.542	S1(10)
			or 24.549	S2(1)
24.583	24.583	24.577	24.583	C(4)
24.621			24.616	S3(0)
			or 24.626	S1(11)
24.677				?, not assigned
24.716		24.711	24.718	S2(2)
			or 24.719	S1(12)
24.779			24.793	S1(13)
24.825	24.824	24.824	24.823	C(5)
24.871		24.868	24.871	S2(3) or S3(2)
			or 24.879	S1(14)
24.968			24.961	S1(15)
25.000			24.995	S3(3)
		or 25.014	25.015	S2(4)
25.062	25.062	25.060	25.060	C(6)
25.116			25.116	S3(4)
25.160		25.158	25.160	S2(5)
25.231			25.234	S3(5)
^14^N_2_^+^				

*The energy values are indicated in eV. The last column lists the proposed ^14^N_2_^+^ state assignments. The S1, S2, and S3 labels refer to the vibrational progressions as identified by Yoshii et al. and “C” refers to the ${\text{C}}^{2}{\text{Σ}}_{u}^{+}$ state of ^14^N_2_^+^. The numbers in brackets are the vibrational quantum numbers*.

All of the features listed in Table 1 are observed in Figure 2 as coincidences between threshold electrons and ^14^N^+^ ions, while the ^14^N_2_^+^ signals, if any, cannot be distinguished from the noise level, with the exception of faint ^14^N_2_^+^ contributions at the location of the C, v′ = 3 and v′ = 4 levels. In other words, above the dissociation threshold at 24.2878 eV, less than about 5% of the ^14^N_2_^+^ produced at the peak positions in the spectrum will survive as molecular ions during the time of about 3 μs that the parent ion spends in the ion acceleration region.

The TPEPICO spectrum obtained when ^15^N_2_ is used as a target is illustrated by Figure 3. Contrary to the absence of significant ^14^N_2_^+^ signals in Figure 2, the data obtained with ^15^N_2_ show several clear molecular ion peaks above the ^15^N^+^ + ^15^N threshold at 24.2927 eV. Because of the neutral's lower zero-point energy, this limit lies 4.9 meV higher than the 24.2878 eV value for ^14^N^+^ + ^14^N.

The peak energies indicated for the vibrational levels v′ = 3–8 of the ^15^N_2_^+^ C-state agree within 3 meV with the ionization energies in brackets, obtained from the C → X emission bandheads for ^15^N_2_^+^ published by Joshi (1966a), and our evaluation of the rotationless ^15^N_2_^+^ (X, v′ = 0)←^15^N_2_ (X, v = 0) ionization energy as 15.5810 eV. Contrary to the ^14^N_2_^+^ C-state, the heavier isotopolog gives rise to clearly distinguished production of ^15^N_2_^+^ for the vibrational levels v′ = 3, 4, and 5, and to a lesser extent also for v′ = 6 and 7. The corresponding branching ratios will be discussed in section Discussion. Between 25.25 and 25.35 eV the enhanced “noise” in the ^15^N_2_^+^ coincidence count hints at the possibility of weak decay into molecular ions. Within the limited time of access to the DESIRS beamline, the reproducibility of this observation could not be verified.

The assignment of the spectral features other than the C-state peaks is based on applying isotope shifts to the progressions labeled S1, S2, and S3 in the 5 meV resolution TPES reported by Yoshii et al. (1997). Vibrational parameters w_e_ and w_e_x_e_ for ^14^N_2_^+^ were obtained from Birge-Sponer plots of the vibrational spacings in the energy range of interest, and these were used to calculate the vibrational isotope shifts according to:

(4) $$\begin{array}{l} \text{G}{\left(\text{v}\right)}_{15}-\text{G}{\left(\text{v}\right)}_{14}={\left(\text{v}+1/2\right)}^{*}{\text{w}}_{\text{e}}^{*}\left[\surd \left(7/7.5\right)-1\right]\\ \;-{\left(\text{v}+1/2\right)}^{2}{\text{w}}_{\text{e}}{\text{x}}_{\text{e}}^{*}\left(7/7.5-1\right), \end{array}$$

where G(v) stands for the vibrational energy of level v with respect to the minimum of the potential well. These shifts were applied to the experimental ^14^N_2_^+^ energy levels of Yoshii et al. (1997), and the ^15^N_2_ ionization energies were obtained by allowing for the lowering of the neutral N_2_ zero-point energy. The energies for the S1, S2, and S3 vibrational levels obtained in this manner are indicated in the upper part of Figure 3. All of the structures observed could thus be attributed to either the C-state or to one of the S1 or S2 or S3 progressions, with the exception of the peaks at 24.650, 25.026, and 25.300 eV which are as of yet unassigned.

## Discussion

In what follows we shall first discuss the branching ratios for decay of the N_2_^+^ C-state levels, and subsequently address the spectroscopic information pertaining to the S1 progression.

### Branching Ratios for Decay of the N_2_^+^ (${\text{C}}^{2}{\text{Σ}}_{\text{u}}^{+}$, v′) Levels

The vibrational levels v′ ≤ 2 of the C-state decay only by fluorescence to the electronic ground state: N_2_^+^ (${\text{C}}^{2}{\text{Σ}}_{\text{u}}^{+}$, v′) → (${\text{X}}^{2}{\text{Σ}}_{\text{g}}^{+}$, v″). Their lifetime is about 79 ns (Erman, 1976), and the higher, predissociated levels necessarily have a shorter lifetime. The timescale for ion detection in the present experiments being of the order of microseconds, the competition between predissociation and fluorescence can therefore be evaluated directly from the relative yields for N^+^ and N_2_^+^ resulting from photoionization into a specific N_2_^+^(C, v′) level.

The corresponding branching ratios were determined by integrating the areas I(N^+^) and I(N_2_^+^) under the C-state N^+^ and N_2_^+^ peaks in the TPEPICO scans. The results are shown in Table 2 for ^14^N_2_^+^ and in Table 3 for ^15^N_2_^+^. They are expressed as branching ratios for N_2_^+^ production, as defined by equation 2, for each of the vibrational levels examined. The competition between C-state predissociation and C → X fluorescence has previously been quantified by comparing measured C → X fluorescence cross sections with those expected in the absence of predissociation. For this purpose, vibrationally resolved fluorescence intensities were measured upon impact of electrons (van de Runstraat et al., 1974) or ions (Govers et al., 1975) on a static room-temperature target of N_2_, at impact speeds high enough for the excitation to the various C-state vibrational levels to be considered as a “vertical” Franck-Condon process. Summing fluorescence from a particular (C, v′) level to all vibrational levels v″ of the N_2_^+^ ground-state, one obtains the vibrational fluorescence cross sections, σ_em_ (v′), and one can write:

(5) $$\begin{array}{l} \sigma_{\text{em}}({\text{v}}^{\prime })=\sigma_{\text{exc}}{\left({\text{v}}^{\prime }\right)}^{*}\text{BR}({\text{v}}^{\prime },{\text{N}}_{2}^{+}), \end{array}$$

where σ_exc_ (v′) is the excitation cross-section for populating the vibrational level under consideration. Using cross-sections relative to those for v′ = 2, for which the BR(v′ = 2, N_2_^+^) is unity, since predissociation is energetically forbidden, one readily obtains the v′ ≥ 3 branching ratios from the relative emission intensities, provided the excitation ratios are known:

(6) $$\begin{array}{l} \text{BR}\left({\text{v}}^{\text{′}},{\text{N}}_{2}^{+}\right)=\left[{\text{σ}}_{\text{em}}\left({\text{v}}^{\prime }\right)/{\text{σ}}_{\text{em}}\left({\text{v}}^{\prime }=2\right)\right]/\left[{\text{σ}}_{\text{exc}}\left({\text{v}}^{\prime }\right)/{\text{σ}}_{\text{exc}}\left({\text{v}}^{\prime }=2\right)\right]. \end{array}$$

The excitation ratios were obtained from the N_2_^+^ (C, v′)←N_2_ (v = 0) Franck-Condon factors, (“FCF”) including configuration interaction (“CI”) (see van de Runstraat et al., 1974; Govers et al., 1975 for details).

**Table 2:** Branching ratios for the decay into stable ^14^N_2_^+^ of the vibrational levels v′ = 2–8 of the ${\text{C}}^{2}{\text{Σ}}_{\text{u}}^{+}$, state of ^14^N_2_^+^.

**Line**	**^**14**^N_2_^+^ (C, v^**′**^)**	**2**	**3**	**4**	**5**	**6**	**7**	**8**
1	BR(^14^N_2_^+^) present work	100%	3.0 ± 0.8%	1.8 ± 0.9%	≤0.3%	≤0.3%	NM	NM
2	Theor. excit. ratio FCF + CI	1.00	1.62	2.07	2.23	2.11	1.81	1.44
3	Fluoresc. ratio ion impact	1.00	0.15	0.11	0.065	0.04	0.03	0.009
4	BR(^14^N_2_^+^) ion impact fluoresc.	100%	9.3 ± 1.2%	5.3 ± 1.0%	2.9 ± 0.4%	1.9 ± 0.5%	1.7 ± 0.5%	0.26 ± 0.06%
5	BR(^14^N_2_^+^) Auger fluorescence	100%	9.1%	5.2%	2.6%	1.6%	NM	NM
6	A_pred_ (v′)/ A_em_ (v′) present work	0.0	32 (25–44)	55 (36–110)	≥332	≥332	NM	NM
7	A_pred_ (v′)/ A_em_ (v′) ion imp. fluo.	0.0	9.8 (8.5–11.5)	18 (15–22)	33 (29–40)	52 (41–69)	59 (44–89)	159 (95–478)
8	A_pred_ (v′)/ A_em_ (v′) Auger fluo.	0.0	10.0	18	27	61	NM	NM

*The first line lists the values obtained from the TPEPICO spectrum of Figure 2 by dividing the areas of the ^14^N_2_^+^ coincidence peaks by the sum of those for ^14^N^+^ and ^14^N_2_^+^ coincidences. The uncertainty limits are obtained after propagation of the original Poisson distribution of the photoelectron image pixel counts. Lines 2 to 4 summarize the data of Govers et al. (1975). Line 5 lists the branching ratios obtained by Ehresmann et al. (2006). The respective ratios between the probability for predissociation to that for C→X fluorescence are indicated in lines 6–8*.

**Table 3:** Branching ratios for the decay into stable ^15^N_2_^+^ of the vibrational levels v′ = 2–8 of the ${\text{C}}^{2}{\text{Σ}}_{\text{u}}^{+}$ state of ^15^N_2_^+^.

**Line**	**^**15**^N_2_^+^ (C, v^**′**^)**	**2**	**3**	**4**	**5**	**6**	**7**	**8**
1	BR(^15^N_2_^+^) present work	100%	37 ± 3 %	24 ± 0.7 %	12 ± 0.6 %	2.1 ± 0.2%	4.9 ± 0.3%	≤4%
2	Theor. excit. ratio FCF + CI	1.00	1.68	2.22	2.47	2.41	2.13	1.73
3	Fluoresc. ratio ion impact	1.00	0.85 ± 0.04	0.88 ± 0.04	0.59 ± 0.03	0.13 (0.11–0.16)	0.15 (0.13–0.18)	0.05 (0.04–0.08)
4	BR(^15^N_2_^+^) ion impact fluoresc.	100%	51 ± 3.0%	39.6 ± 1.8%	23.9 ± 1.3%	5.4 (4.6–6.6)%	7.1 (6.1–8.5)%	2.9 (2.3–2.6)%
5	A_pred_ (v′)/A_em_ (v′) ion imp. fluo.	0	0.98 ± 0.10	1.53 ± 0.10	3.19 ± 0.20	17.6 ± 3.5	13.3 ± 2.4	34 (21–42)
6	A_pred_ (v′)/A_em_ (v′) present work	0	1.70 ± 0.24	3.17 ± 0.18	7.3 (6.9–7.8)	46 (42–52)	19 (18–21)	≥24

*The first line lists the values obtained from the TPEPICO spectrum of Figure 3 by dividing the areas of the ^15^N_2_^+^ coincidence peaks by the sum of those for ^15^N^+^ and ^15^N_2_^+^ coincidences. The uncertainty limits are obtained after propagation of the original Poisson distribution of the photoelectron image pixel counts. Lines 2 to 4 summarize the data of Govers et al. (1975). Lines 5 and 6 list the respective ratios between the probability for predissociation to that for C→X fluorescence*.

The data for ^14^N_2_^+^ are summarized in Table 2 with the results of the present experiments in the first line. Weak parent ion signals could be distinguished from the background noise only for the levels v′ = 3 and v′ = 4. As a result, only upper limits to the ^14^N_2_^+^ branching ratios are listed for v′ = 5–8. The branching ratios in line 4 are those obtained from the fluorescence spectra observed in the ion-impact experiments (Govers et al., 1975); they are somewhat more precise than the closely similar electron-impact results (van de Runstraat et al., 1974). They are obtained by dividing the measured fluorescence intensities, relative to that of the un-predissociated v′ = 2 level (line 3), by the theoretical excitation ratios obtained by assuming a Franck-Condon excitation with configuration interaction (line 2). Line 5 reproduces the ^14^N_2_^+^ branching ratios obtained by Ehresmann et al. (2006), who analyzed the dispersed C → X fluorescence observed when populating the C-state by photon excitation of the 1s^−1^ π^*^ resonance at energies between 400 and 403 eV, using room-temperature ^14^N_2_ as a target. Lines 6–8 list the ratios between the probability for predissociation to that for C → X fluorescence deduced from the three experiments. The close agreement between the results of Ehresmann et al. (2006) and those summarized in lines 4 and 7 is gratifying, especially considering the difference in the C-state excitation mechanism pertaining to the two types of experiment. It suggests that the excitation ratios in line 2 of Table 2 can be used with reasonable confidence, at least up to v′ = 6.

The fact that that the present measurements yield ^14^N_2_^+^ branching ratios substantially lower than those obtained from the analysis of fluorescence intensities is therefore surprising. We shall see below that in the case of ^15^N_2_^+^ the difference between the two sets of results is less pronounced, so that an experimental artifact can apparently be excluded. The only explanation that we can propose to account for the lower ^14^N_2_^+^ branching ratios concerns the rotational temperature of the target nitrogen, as will be further discussed below. In all predissociation experiments conducted so far, the target gas was static nitrogen at room temperature. In the present experiment it was a supersonic beam in which the rotational distribution is much narrower, as discussed in section Method and witnessed by the small widths of the C-state peaks in Figures 2, 3.

In the case of ^15^N_2_^+^, distinct parent ion peaks were found for the C-state levels v′ = 3, 4, and 5, weak peaks for v′ = 6 and 7, while only an upper limit could be estimated for v′ = 8. The present branching ratios are summarized in line 1 of Table 3. The present ^15^N_2_^+^ branching ratios shown in line 1 decrease with increasing vibration in a manner quite similar to the fluorescence data, listed in line 4. They show a systematic trend toward lower ^15^N_2_^+^ production, that is, higher predissociation rates. The corresponding ratios of the rate of predissociation to that of fluorescence are listed in lines 5 and 6.

The fluorescence rate of an electronically excited state is not expected to vary strongly with vibrational quantum number or isotopic substitution, as these parameters to a first approximation affect only the nuclear motion. The strong variation of the A_pred_ (v′)/A_em_ (v′) ratios in Tables 2, 3 is therefore essentially due to changes in the rate of predissociation (van de Runstraat et al., 1974 and references therein).

Two different models have been considered to account for the vibrational dependence of the C-state predissociation rate, and its variation upon isotopic substitution. The first is the accidental predissociation model proposed by Lorquet and Desouter (1972) and further discussed by Lorquet and Lorquet (1974). It incorporates a critical dependence on the energy match between the two interacting bound states. These authors focused on accounting for the observed vibrational and isotopic dependences and did not explicitly consider rotational effects. But as the zero-order energy match will vary with rotation if the two interacting bound states have different rotational constants, rotational effects are quite conceivable within the frame of their accidental predissociation model.

The second model is that of the direct predissociation by the continuum of the B state (see Figure 1) proposed by Tellinghuisen and Albritton (1975) and further detailed by Roche and Tellinghuisen (1979). They correctly reproduced the observed vibrational and isotopic dependencies and also explicitly examined the effect of rotation. It was shown that for ^15^N_2_^+^ the predissociation rate at low N quantum number increases markedly with decreasing rotation. Rotationally cold (^15^N_2_^+^, C) ions produced by photoionization of a supersonic nitrogen beam, as is the case in the present work, will, according to that analysis, predissociate faster than those produced by ionization of a static room-temperature target, in agreement with the tendency summarized in Table 3. However, Roche and Tellinghuisen (1979) also showed that for ^14^N_2_^+^ the rotational dependence of the predissociation rate is weak, which should lead to only a small difference between the fluorescence- and coincidence results, contrary to what is seen in Table 2.

Subsequent theoretical investigations have not yet elucidated the preponderance of accidental predissociation or of direct predissociation in the C-state decay. Langhoff and Bauschlicher (1988) carried out very accurate calculations of the N_2_^+^ doublet states and also of the potential energy crossings between the C ^2^${\text{Σ}}_{\text{u}}^{+}$ state and close-lying ${}^{2}{\text{Σ}}_{\text{u}}^{-}$ and ${}^{4}{\text{Π}}_{\text{u}}$ states. They proposed predissociation of the C-state to occur by spin-orbit coupling to the ${}^{2}{\text{Σ}}_{\text{u}}^{-}$ state followed by transition to the continuum of the ${}^{4}{\text{Π}}_{\text{u}}$ state, in accordance with the model of Lorquet and Desouter (1972). Hochlaf et al. (1997) showed that another quartet state, e ${}^{4}{\text{Σ}}_{\text{u}}$, crosses the C-state near the v′ = 3 vibrational level. It correlates with ground-state atomic fragments and offers a pathway for direct predissociation by spin-orbit coupling. More recently, Paulus et al. (2016) published a time-dependent description of the ^14^N_2_^+^ C-state predissociation through non-adiabatic coupling with the B-state continuum, and they reported predissociation rates that agree quite well with those deduced from the analysis of the C → X fluorescence spectra.

We note that the characteristics of the competition between fluorescence and predissociation, and in particular its dependence on isotopic substitution, are rather unique to the N_2_^+^ C-state, as other states in the vicinity of the He^+^ recombination energy are fully predissociated for both isotopologues investigated here. This supports the analysis of the isotope effects observed in near-thermal charge transfer between He^+^ and N_2_, whereby it was assumed that it results from the sole decay characteristics of the C-state (Govers et al., 1974, 1977). This assumption, and the high D′ ${}^{2}{\text{Π}}_{\text{g}}$ → A ${}^{2}{\text{Π}}_{\text{u}}$ emission intensities observed in low-pressure charge-transfer experiments, indicate that about 90% of the ^14^N_2_^+^ product ions result from initial charge transfer into the D′ ${}^{2}{\text{Π}}_{\text{g}}$ state discussed below (Sekiya et al., 1987; Govers, 2016). This reaction is exothermic by <0.9 eV and does not impart to the neutral He the 2.5 eV kinetic energy necessary to escape from the earth's attraction. The only sufficiently exothermic channel identified so far, i.e., charge transfer into the N_2_^+^ (${\text{B}}^{2}{\text{Σ}}_{\text{u}}^{+}$, v′ ≤ 5) levels, has a rate constant of the order of 1 to 2.10^−11^ cm^3^/s (Govers et al., 1977). This is too small a rate to contribute significantly to the loss of He from the earth's atmosphere (see Lie-Svendsen et al., 1992).

### Spectroscopy of the S1 Progression

The long vibrational progression labeled S1 by Yoshii et al. (1997) has been identified as resulting from photoionization to the second N_2_^+^ state of ${}^{2}{\text{Π}}_{\text{g}}$ symmetry by Baltzer et al. (1992). It was noted that the energy spacings between its lower vibrational levels is smaller than that of the higher ones, as can be understood from the unusual shape of the potential well illustrated in Figure 1, which results from avoiding crossings with two other ${}^{2}{\text{Π}}_{\text{g}}$ states. The intensities of the 2 ${}^{2}{\text{Π}}_{\text{g}}$(v′ ≤ 2) peaks are low, and extracting the adiabatic ionization potential from the (T)PES spectra is rather uncertain.

In the above analyses of the photoelectron spectra, no use was made of the results obtained by Cossart et al. (1985), who analyzed the previously unidentified emission between 229 and 245 nm resulting from low-energy collisions between He^+^ and N_2_ (Holland and Maier, 1971; Govers et al., 1977). Using a novel discharge source and photographic recording, they carried out a rotational analysis complemented by SCF ab-initio calculations, and assigned the most prominent of these emissions to the transition D′ ${}^{2}{\text{Π}}_{\text{g}}$(v′) → A ${}^{2}{\text{Π}}_{\text{u}}$ (v″ = 7, 8, and 9). The emitting D′ ${}^{2}{\text{Π}}_{\text{g}}$(v′) vibrational levels were tentatively labeled as v′ = 0, 1, 2. They are located at 23.698, 23.786, and 23.870 eV above the ground state of ^14^N_2._ There should be only two states of ${}^{2}{\text{Π}}_{\text{g}}$ symmetry in this energy region (Thulstrup and Andersen, 1975), so that the 2 ${}^{2}{\text{Π}}_{\text{g}}$ state identified in the photoelectron spectra and the D′ ${}^{2}{\text{Π}}_{\text{g}}$ state identified by Cossart et al. (1985) must be one and the same state of N_2_^+^.

Accordingly, we have re-examined the published (T)PES data for the D′ ${}^{2}{\text{Π}}_{\text{g}}$ state while locating the first three vibrational levels at the energy values deduced from the analysis by Cossart et al. (1985). For the energies of the levels v′ = 3 to 10 we used the averages of those determined by (T)PES: Baltzer et al. (1992), Yoshii et al. (1997), Yencha et al. (2014) and this work. From the vibrational energy spacings thus obtained, the vibrational parameters w_e_ and w_e_x_e_ were deduced from the intercept and slope of the Birge-Sponer plot:

(7) $$\text{G}({\text{v}}^{\prime }+\text{1})-\text{G}({\text{v}}^{\prime })={\text{w}}_{\text{e}}-{2\text{w}}_{\text{e}}{\text{x}}_{\text{e}}{}^{*}({\text{v}}^{\prime }+1)$$

The energy of the ^14^N_2_^+^ D′ ${}^{2}{\text{Π}}_{\text{g}}$(v′ = 9) level was fixed at 24.4582 eV, the average value obtained by (T)PES, with an agreement within 1 meV between the four sets of experimental data. The resulting least-squares fit yielded w_e_ = 85.53 meV and w_e_x_e_ = 0.085 meV, and T_e_ = 23.6533 eV (potential minimum above the neutral's ground state).

The vibrational levels for v′ ≥ 10 are not well reproduced by the vibrational parameters quoted above. Using equation 7, a least-squares fit to the averaged ^14^N_2_^+^ data obtained by Baltzer et al. (1992), Yoshii et al. (1997), Yencha et al. (2014) and ourselves, yielded w_e_ = 94.20 meV and w_e_x_e_ = 0.085 meV, and T_e_ = 23.6533 eV.

For the ^15^N_2_^+^ D′ ${}^{2}{\text{Π}}_{\text{g}}$ (v′ = 0–10) levels, the vibrational parameters estimated by correcting for the reduced mass were w_e_ = 82.63 meV and w_e_x_e_ = 0.079 meV, respectively. Allowing for the lowering of the neutral ground state by 0.59 meV, the ionization energies for the first eleven vibrational levels were predicted as indicated in Table 4, which shows a satisfactory agreement with the v′ = 7–10 energies observed in in the present experiments.

**Table 4:** Predicted and exptl. ionization energies ^15^N_2_^+^ D′ ${}^{2}{\text{Π}}_{\text{g}}$ (v′ = 0–10) in eV w_e_ = 82.63 meV, w_e_x_e_ = 0.079 meV; T_e_ = 23.6583 eV.

**v^**′**^**	**These expts**.	**Predict**	**Expt.-pred**.
0		23.700	
1		23.782	
2		23.864	
3		23.946	
4		24.028	
5		24.110	
6		24.192	
7	24.273	24.274	−0.001
8	24.360	24.355	0.005
9	24.430	24.436	−0.006
10	24.518	24.517	0.001
		^15^N_2_^+^	

*Vibrational parameters and ionization energies predicted from these parameters for the vibrational levels v′ = 0–10 of the D′ ${}^{2}{\text{Π}}_{\text{g}}$ of ^15^N_2_^+^. The vibrational parameters were obtained by applying the reduced-mass correction to those obtained for ^14^N_2_^+^ by a Birge-Sponer fit to the available experimental data. T_e_ situates the bottom of the potential well with respect to the ground-state of neutral ^15^N_2_*.

A separate fit to the present peak positions in Figure 3 was carried out for the levels v′ ≥ 10. The results are listed in Table 5.

**Table 5:** Fit to ^15^N_2_^+^ D′ ${}^{2}{\text{Π}}_{\text{g}}$ (v′ = 10–24) in eV w_e_ = 94.20 meV, w_e_x_e_ = 0.545 meV; T_e_ = 23.6583 eV.

**v^**′**^**	**These expts**.	**Fit eV**	**Exp-Fit eV**	**Overlap?**
10	24.518	24.517	0.001	
11	24.591	24.599	−0.008	
12	not observed	24.680		
13	24.746	24.760	−0.015	S3(1)
14	24.855	24.839	0.016	S2(3) S3(2)
15	24.932	24.917	0.015	
16	24.998	24.994	0.004	S2(4) S3(3)
17	25.071	25.070	0.001	
18	25.134	25.144	−0.010	S2(5)
19	25.194	25.218	−0.024	
20	25.272	25.290	−0.018	S2(6)
21	25.358	25.361	−0.004	
22	25.430	25.432	−0.002	S3(7)
23	25.498	25.501	−0.003	
24	25.565	25.569	−0.004	
^15^N_2_^+^				

*Ionization energies and vibrational parameters for the vibrational levels v′ = 10–24 of the D′ ${}^{2}{\text{Π}}_{\text{g}}$ state of ^15^N_2_^+^. The vibrational parameters were obtained by a least-squares Birge-Sponer fit to the present S1 peak energies of Figure 3*.

Comparing the observed peak values and vibrational ionization energies calculated for the ^15^N_2_^+^ D′ ${}^{2}{\text{Π}}_{\text{g}}$ state, we note several cases where the difference somewhat erratically exceeds 10 meV. Yet, the accuracy of the experimental energy scale is of the order of 1 meV, and the narrowest peaks in Figure 3 have a halfwidth of about 10 meV. The differences just mentioned may result in part from overlap with neighboring S2 or S3 states, as seen from the labeling in Figure 3. But they also arise from the shape and/or widths of the peaks observed in the TPEPICO spectra. The peaks at 25.134 eV and at 25.194 eV in Figure 3, for instance, have narrow halfwidths, of the order of 10 to 15 meV, even though they may comprise contributions from two or three different vibrational states. In contrast, the peaks at 24.430 and at 24.932 eV, where no superposition is expected, have halfwidths of the order of 30–40 meV.

Even with a bandpass for threshold electrons as narrow as 0–5 meV, one cannot exclude contributions from electrons resulting from autoionization of nearby Rydberg states. The variation of relative peak intensities depending on the electron energy bandpass can in fact be used to distinguish structures that localize autoionizing neutral states from those that result from resonant ionization to a specific cation state (Bréchignac et al., 2014). That possibility has not yet been exploited in the present investigation. Baltzer et al. (1992), whose HeII PES is not subject to near-resonant autoionization, noted that the PES peaks corresponding to the D′ ${}^{2}{\text{Π}}_{\text{g}}$ state were 30% broader than those of the C-state.

We consider that the N_2_^+^ D′ ${}^{2}{\text{Π}}_{\text{g}}$ (v′) energies exhibit irregularities that at least in part reflect the varying strengths of the interactions that cause these levels to predissociate. The D′ ${}^{2}{\text{Π}}_{\text{g}}$ (v′ ≤ 2) levels, which energetically cannot predissociate and only decay by fluorescence to the A ${}^{2}{\text{Π}}_{\text{u}}$ state, have rather long lifetimes, 6.10^−7^ s or more, and possibly as large as 10^−5^ s (Govers et al., 1977). For the D′ ${}^{2}{\text{Π}}_{\text{g}}$ (v′ ≥ 8) levels situated above the first dissociation limit, even a very weak interaction with a dissociation continuum will therefore cause predissociation to compete effectively with fluorescence. On the other hand, considering the proximity of the repulsive parts of the D′ ${}^{2}{\text{Π}}_{\text{g}}$ and D ${}^{2}{\text{Π}}_{\text{g}}$ potentials, it may well be that their mutual interaction, already invoked by Baltzer et al. (1992), is at certain energies strong enough to locally enhance direct transition to the D-state continuum, while simultaneously attributing a strong dissociative character to the first-order bound D′ level. Narrow peaks in the N^+^ coincidence spectrum would in this picture result from rather weak predissociation, and the wider peaks from rapid predissociation, concomitant with locally enhanced transitions to the dissociation continuum. But in either case, because the D′ fluorescence rate is so low, predissociation will dominate radiative decay to stable N_2_^+^ so that only N^+^ ions are detected in the TPEPICO spectrum.

Information about the lifetime of predissociated molecular ions can be obtained from the asymmetry of the fragment ion time-of-flight peaks (Baer and Tuckett, 2017), provided that the parent ion fragments within the 3 μs it takes to exit the acceleration regions. A preliminary analysis suggests lifetimes of the order of a few microsecond for several of the features (other than those of the C-state vibrational levels) present in the TPEPICO spectra of Figures 2, 3. A more systematic investigation of such “metastable ions” decay will be discussed in a forthcoming publication.

## Conclusions

The direct measurement of the branching ratios between molecular ions and atomic fragments shows that predissociation of N_2_^+^ dominates its unimolecular decay as soon as the photoionization energy surpasses the first dissociation limit. The sizable decay by fluorescence of the N_2_^+^ (${\text{C}}^{2}{\text{Σ}}_{\text{u}}^{+}$, v′ ≥ 3) levels appear to be a rather unique exception, and the present TPEPICO measurements support the conclusions of earlier fluorescence measurements as to the effect of vibrational excitation and isotopic substitution on the rate of predissociation. The differences noted between the results of the two experiments suggest that rotational excitation may significantly modify the rate of predissociation also. This could be verified by repeating the TPEPICO experiments using an effusive beam, rather than a supersonic beam as photoionization target. It is hoped that such additional data on rotational effects, including those for the mixed ^14^N^15^N^+^ isotopolog, will stimulate further theoretical work detailing the mechanism of accidental- and/or direct predissociation of the ${\text{C}}^{2}{\text{Σ}}_{\text{u}}^{+}$ state.

## Author Contributions

All authors listed have made a substantial, direct and intellectual contribution to the work, and approved it for publication.

### Conflict of Interest Statement

The authors declare that the research was conducted in the absence of any commercial or financial relationships that could be construed as a potential conflict of interest. The reviewer IF declared a past co-authorship with one of the authors GG to the handling editor.
